# Evidence of timing effects on acupuncture: A functional magnetic resonance imaging study

**DOI:** 10.3892/etm.2014.2056

**Published:** 2014-11-07

**Authors:** YANLING GAO, ZHICHENG LIN, JING TAO, SHANLI YANG, RI CHEN, CAI JIANG, KENG DONG, JIA HUANG, LIDIAN CHEN

**Affiliations:** 1 Department of Rehabilitation Medicine, Rehabilitation Hospital, Fujian University of Traditional Chinese Medicine, Fuzhou, Fujian 350001, P.R. China; 2 College of Rehabilitation Medicine, Fujian University of Traditional Chinese Medicine, Fuzhou, Fujian 350108, P.R. China; 3 Department of Rehabilitation Medicine, The First Hospital of Quanzhou, Quanzhou, Fujian 362000, P.R. China; 4 Fujian Provincial Rehabilitation Engineering Research Center, Fujian University of Traditional Chinese Medicine, Fuzhou, Fujian 350108, P.R. China

**Keywords:** acupuncture, timing effect, stroke, functional magnetic resonance imaging

## Abstract

According to Traditional Chinese Medicine there is an optimum time to administer acupuncture at a particular acupoint. The present study used functional magnetic resonance imaging to investigate the timing effects of acupuncture at the Zusanli (ST36) acupoint. A total of 10 healthy volunteers and 10 post-stroke patients were recruited. The subjects received acupuncture stimulation at ST36 during two time periods: between 7:00 a.m. and 9:00 a.m. (the AM condition) and between 3:00 p.m. and 5:00 p.m. (the PM condition), seven days later. Blood oxygenation level-dependent signals were captured while the patient was receiving the acupuncture stimulation. The results showed a stronger activation in the AM condition than in the PM condition in both healthy and stroke subjects. The significant regions in the healthy subjects included the prefrontal cortex, cingulum, thalamus and cerebellum; for the stroke patients, the significant regions were the cuneus, supplementary motor area and inferior parietal gyrus. Timing can therefore modulate brain activation patterns during acupuncture in healthy subjects and stroke patients; however, the modulation effect appears to differ between the two subject groups. Further studies are required to explore the timing effects of acupuncture at different acupoints in different populations.

## Introduction

Acupuncture has a long history in treating various diseases and physiological malfunctions in Eastern medicine and is gaining increasing popularity worldwide (1,2). According to a review by the World Health Organization Consultation on Acupuncture, stroke ranked highly among the ailments for which acupuncture had proved to be effective (3). In addition, acupuncture is effective in a broad spectrum of diseases and disorders, and is therefore regarded as a nonspecific therapy in clinical practice (4). It is important to understand the mechanism of acupuncture within the context of Traditional Chinese Medicine (TCM). According to the principles of TCM, ‘qi’ is an important constituent of the human body that is transported by the meridian system (5). The meridian system is a complex network of neurovascular bundles that are characterized by distributed conductance, resistance, inductance and capacitance as functions of time and frequency (6). In other words, qi interacts with time and flows through the body. Based on this model, acupuncture works by regulating the circulation of qi (7). Given the relationship among qi, time and meridian, the method of Zi-Wu-Liu-Zhu is useful in calculating the optimum time to administer acupuncture at a particular meridian (8). For example, qi is strongest on the stomach meridian of Foot-Yangming between 7:00 a.m. and 9:00 a.m., which indicates that the optimum time for acupuncture at the Foot-Yangming is between 7:00 a.m. and 9:00 a.m. (9). Converging evidence from animal studies (10,11) and clinical investigations (12–14) indicates that the time to administer acupuncture determines the effectiveness and success of the procedure. In the last five years, time-related acupuncture has received increased focus and has been developed as an interdiscipline of chronoacupuncture (8).

It has been suggested that the majority of acupuncture effects are mediated via the brain (15). With the development of neuroimaging techniques, functional magnetic resonance imaging (fMRI) is becoming a useful method to investigate the neural mechanism of acupuncture. An increasing number of fMRI studies have demonstrated different brain networks for acupuncture performed at different acupuncture points (16). Even for a particular acupuncture point, differences were revealed between healthy subjects and patients (17,18). To the best of our knowledge, the neural mechanism underlying the effect of timing on acupuncture has not been investigated. The present study aimed to investigate brain activation patterns during acupuncture at different time periods and to consider the timing effects in stroke patients and healthy subjects. The acupuncture point of Zusanli (ST36), which has been frequently used in the treatment of stroke patients in clinical practice in China (17,19,20), was selected. In the literature, acupuncture stimulation at ST36 in healthy individuals demonstrated effects in the somatosensory (5,21) and motor (21–23) areas, cerebellum (5,17,24), limbic system (17,21,23–28) and higher cognitive areas (5,17,21,23,28). Compared with healthy controls, a weaker activation pattern of a similar network was revealed for stroke patients (17). In the present study the timing effect was tested by comparing acupuncture at the optimum time of between 7:00 a.m. and 9:00 a.m. (the AM condition) and a non-optimum time of between 3:00 p.m. and 5:00 p.m. (the PM condition). It was hypothesized that acupuncture stimulation at ST36 would differ during the time periods of 7:00–9:00 a.m. and 3:00–5:00 p.m., and the timing effect would differ between stroke patients and healthy subjects.

## Materials and methods

### Subjects

The patient group was composed of 10 patients (five males and five females; mean age, 58.10±9.34 years) with subcortical ischemic stroke. The patients suffered from their first-ever stroke >6 months prior to the enrollment. Descriptive data for the patient group are summarized in Table I. The control group was composed of 10 healthy volunteer subjects (five males and five females; mean age, 56.0±9.19 years). All subjects were right-handed and had no acupuncture therapy experience. They did not have any history of psychiatric or neurological disorders. The present study protocol was approved by the Ethics Committee of Fujian University of Traditional Chinese Medicine (Fuzhou, China). All subjects provided written informed consent in accordance with the Medical Ethics Committee of Fujian University of Traditional Chinese Medicine (2013KY-004-02).

### fMRI task design

**Figure 1:**
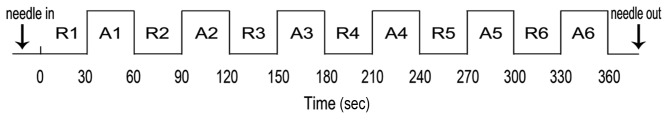
Experimental paradigm of the block design. R, rest block; A, acupuncture block.

The acupoint of ST36 is located on the tibialis anterior muscle, four fingerbreadths below the lower margin of the patella and one fingerbreadth across from the anterior crest of the tibia (25). Manual acupuncture manipulations at ST36 on the left leg were performed by the same experienced and licensed acupuncturist. The stainless silver needle was 0.3 mm in diameter and 40 mm in length. The needle was inserted into the skin surface of ST36 at a depth of 1.0–2.0 cm with a frequency of 2 Hz. ‘De qi’, a composite of unique sensations (29), was to be achieved through needle manipulation. The stimulation condition was alternated with the rest condition in a block design, and each condition lasted 30 sec in duration (Fig. 1). To ensure control was maintained for the duration of 30 sec, the acupuncturist received instructions via audio signal through headphones. In total, 12 blocks (six acupuncture blocks and six rest blocks) were performed within a single session. All subjects were scanned twice, once between 7:00 a.m. and 9:00 a.m. and again between 3:00 p.m. and 5:00 p.m. The sequence of the two sessions was randomized across the subjects. To avoid potential long-lasting effects of acupuncture (30,31), the two sessions were applied with an interval of seven days. Following each scan, the feeling of ‘de qi’ was assessed and checked for in each subject by interview.

### fMRI acquisition

The fMRI series was acquired by a 3-T MRI machine (GE HDXT; GE Healthcare Bio-Sciences, Pittsburgh, PA, USA). The subjects were asked to adopt a supine position and instructed to lie quietly and to keep their eyes closed. Foam cushions were used to minimize head movement, and earplugs were used to reduce noise interference. The functional T2^*^ images were obtained with an echo planar imaging sequence using the following parameters: Repetition time (TR)/echo time (TE), 3,000 msec/40 msec; flip angle, 90°; field of view (FOV), 240×240 mm. Structural T1-weighted images were obtained with a magnetization-prepared rapid gradient echo sequence using the following parameters: TR/TE, 2,000 msec/24 msec; FOV, 240×240 mm.

### fMRI data analysis

The fMRI data were analyzed using Statistical Parametric Mapping (SPM) 8 (Wellcome Department of Imaging Neuroscience, London, UK) and implemented in MATLAB^®^ (MathWorks, Natick, MA, USA). To avoid the nonequilibrium effects of magnetization, the first eight scans were discarded. Preprocessing included realignment of the functional time series to correct for head movement, and subjects with head motion of >2.5 mm of translation or >2.5° of rotation in any direction were excluded from this study. The resulting images were further spatially normalized to the standard Montreal Neurological Institute (MNI) space and were smoothed with a Gaussian kernel of 6 mm (full-width half-maximum).

At the individual level, the preprocessed data were fitted to a general linear model in SPM 8 (32) using a box-car function. The contrast of interest (acupuncture-rest) was obtained in each subject on AM and PM occasions. In order to explore the timing effect on acupuncture, the following contrast was also conducted at the individual level to control for the effect of repeated measurements (33,34): (acupuncture_AM_-rest_AM_) - (acupuncture_PM_-rest_PM_) and (acupuncture_PM_-rest_PM_) - (acupuncture_AM_-rest_AM_). At the group level, whole brain analyses of one-sample t-tests with random effects were performed (35). The statistical threshold for the t-images was P<0.001 (uncorrected) at the voxel level, with a cluster size of 20 voxels. Finally, all of the activation maps were projected to MNI space for the identification of regions involved in the contrasts of interest. The ‘Local Maxima Labeling’ embedded in the automated anatomical labeling method was used to label the peak coordinates of the blood oxygenation level-dependent (BOLD) clusters (36).

## Results

### Brain activations of ST36

**Figure 2:**
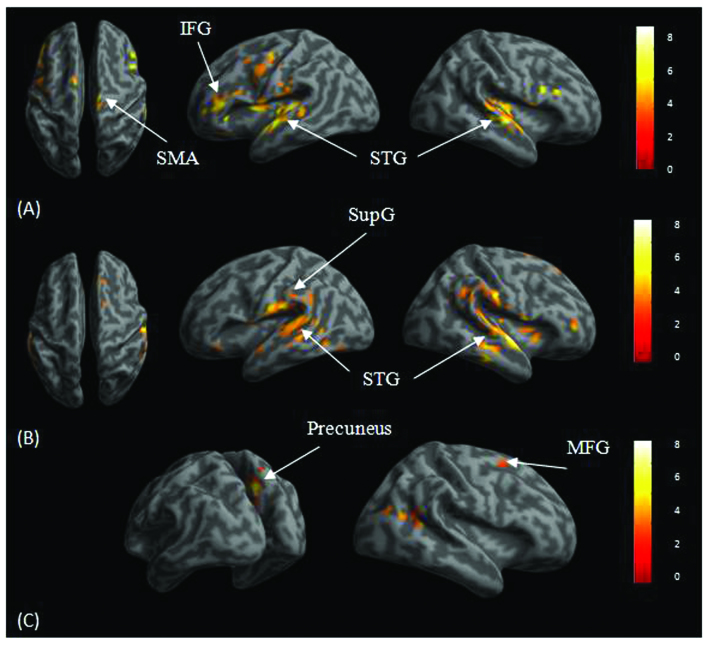
Significant brain activations in the (A) healthy subjects at the AM condition; (B) stroke subjects in the AM condition; and (C) stroke subjects in the PM condition. The threshold was P<0.001 (uncorrected) at the voxel level with cluster size >20 voxels. SMA, supplementary motor area; IFG, inferior frontal gyrus; STG, superior temporal gyrus; SupG, supramarginal gyrus; MFG, middle frontal gyrus.

For the healthy subjects, group analyses on the AM condition revealed activations in the bilateral superior temporal gyrus, left hippocampus, bilateral inferior frontal gyrus, left middle frontal gyrus and bilateral supplementary motor area (Fig. 2). Activations were revealed in the right superior temporal gyrus in the PM condition. For the stroke subjects, results for the AM condition showed activations in the bilateral superior temporal gyrus, right middle frontal gyrus, right superior frontal gyrus, left supramarginal gyrus, left putamen, left amygdala, right supplementary motor area and occipital regions, including the cuneus and calcarine. Results in the PM condition showed activations in the right precuneus, right middle temporal gyrus and right middle frontal gyrus.

### Timing effect

Group analysis for the healthy subjects revealed stronger activations in the right middle frontal gyrus, right superior frontal gyrus, left cingulum, right thalamus and right cerebellum in the AM condition than those in the PM condition (Table II). For the stroke subjects, the results revealed stronger activations in the left cuneus, right supplementary motor area and right inferior parietal gyrus in the AM condition than those in the PM condition.

## Discussion

The present study investigated brain activation patterns in healthy and stroke subjects receiving acupuncture at the acupuncture point of ST36 during two time periods, one in the morning (the AM condition) and one in the afternoon (the PM condition). The main finding was a stronger activation in the AM than in the PM condition in both subject groups. To the best of our knowledge, this is the first neuroimaging report providing evidence for the mechanism of chronoacupuncture.

Acupuncture of ST36 has consistently been shown to elicit reduced neural activity in the limbic system (24,37). Similar limbic reductions in neural activity have also been found with other acupuncture points, such as LI4 (38), LV3 (39) and ST44 (23). These findings suggest a general modulation effect of acupuncture on the limbic system. Given the widespread anatomical and functional connections of the limbic system (40), it is expected that acupuncture affects the limbic system. The present results for the AM condition revealed activations of the hippocampus in the healthy subjects and putamen in the stroke subjects. The reversed pattern of BOLD response could be attributed to different control conditions. The studies mentioned previously used tactile stimulation as the baseline condition (24,37), whereas the present study used the resting state as the control condition. In addition to limbic activations, the present results showed activations for the AM condition in the superior temporal gyrus, prefrontal cortex and supplementary motor area in both healthy and stroke subjects. Prefrontal activations have been found previously in healthy (37) and stroke (41) subjects, suggesting possible higher cognitive modulation effects. This is in agreement with the theory that the cerebro-cerebellar system (42) could account for acupuncture effects.

Notably, the present results revealed significantly different activations following acupuncture of ST36 between the AM and PM conditions. Stronger activations were found in the middle and superior frontal gyrus, cingulum, thalamus and cerebellum in the AM condition than those in the PM condition in healthy subjects. Similar results of stronger activations in the AM condition than those in the PM condition were revealed for the stroke subjects: the regions included the left cuneus, right supplementary motor area and right inferior parietal gyrus. The results were in agreement with the theory of chronoacupuncture stating that there is an optimum time for acupuncture to achieve the most curative effect (8), and this appears to apply to both healthy subjects and stroke patients. The factor of timing in conducting acupuncture has been largely ignored in previous neuroimaging studies, which may have contributed to their heterogeneous results (16,43) and low test-retest reliability (44). Although the factors of acupuncture points, acupuncture manipulation and stimulation methods have been investigated, the consideration of timing in conducting acupuncture is also important.

The present results demonstrated different fMRI activation patterns of acupuncture at ST36 at different time periods in stroke patients and healthy subjects. Future studies with larger sample sizes should further explore the timing effects of acupuncture at different acupoints and in different populations.

## Figures and Tables

**Table I:** Demographics of the stroke subjects.

				Ischemic stroke		
				
Patient no.	FM-UL	Gender	Age (years)	Side	Area	Days after stroke
1	16	M	45	R	Basal ganglia, internal capsule	221
2	12	M	66	R	Basal ganglia, internal capsule, corona radiate	195
3	14	F	70	R	Basal ganglia, internal capsule	243
4	13	F	57	R	Basal ganglia, internal capsule, corona radiate	186
5	15	F	42	R	Basal ganglia, internal capsule	210
6	11	M	69	R	Basal ganglia, internal capsule	218
7	10	F	54	R	Basal ganglia, internal capsule, corona radiate	237
8	12	M	59	R	Basal ganglia, internal capsule	204
9	9	M	67	R	Basal ganglia, internal capsule, corona radiate	188
10	15	F	52	R	Basal ganglia, internal capsule	242

FM-UL, lower limb section of the Fugl-Meyer Scale; M, male; F, female; R, right.

**Table II:** Summary of significant brain activations for the longitudinal contrast of (AM-PM) and (PM-AM) during acupuncture at the left ST36 in the healthy and patient groups.

A, Healthy group (n=10)				

Coordinates x, y, z (mm)	L/R	Label	Cluster size (voxels)	T
AM>PM				
42, 36, 18	R	Middle frontal gyrus	1076	8.93
6, 39, 45	R	Superior frontal gyrus	777	8.03
−12, −39, 18	L	Posterior cingulum	91	7.68
9, −42, −15	R	Cerebellum	403	7.32
9, −30, 21	R	Thalamus	88	7.26
3, −21, −30	R	Cerebellum	67	6.35
PM>AM				
NA				

B, Stroke group (n=10)				

Coordinates x, y, z (mm)	L/R	Label	Cluster size (voxels)	T

AM>PM				
3, −84, 39	L	Cuneus	84	9.40
9, 21, 45	R	Supplementary motor area	95	6.42
54, −54, 42	R	Inferior parietal gyrus	89	5.21
PM>AM				
NA				

The thresholds were P<0.001 (uncorrected) at the voxel level, P<0.05 (familywise error corrected) at the cluster level. T, T-values of activation foci; R, right; L, left; NA, not applicable.

## Abbreviations

BOLD: blood oxygenation level-dependent
fMRI: functional magnetic resonance imaging
FM-UL: lower limb section of the Fugl-Meyer Scale
IFG: inferior frontal gyrus
MFG: middle frontal gyrus
MNI: Montreal Neurological Institute
SMA: supplementary motor area
STG: superior temporal gyrus
SupG: supramarginal gyrus
TCM: Traditional Chinese Medicine
